# HIV transmission among acutely infected participants of a Dutch cohort study 2015–2021 is not associated with large, clustered outbreaks

**DOI:** 10.1097/QAD.0000000000003416

**Published:** 2022-11-18

**Authors:** Henrieke A.B. Prins, Casper Rokx, Annelies Verbon, Ard van Sighem, Godelieve J. de Bree, Maartje Dijkstra, Jan M. Prins, Peter Reiss, Jeroen J.A. van Kampen, David A.M.C. van de Vijver

**Affiliations:** a Department of Medical Microbiology and Infectious Diseases and Department of Internal Medicine, Erasmus University Medical Centre, Rotterdam, The Netherlands; b Stichting HIV Monitoring (SHM), Amsterdam, The Netherlands; c Department of Internal Medicine, Division of Infectious Diseases, and Amsterdam Institute for Infection and Immunity, Amsterdam UMC, University of Amsterdam; d Department of Infectious Diseases, Public Health Service of Amsterdam; e Department of Global Health, Amsterdam UMC, University of Amsterdam, and Amsterdam Institute for Global Health and Development, Amsterdam; f Department of Viroscience, Erasmus University Medical Centre, Rotterdam, the Netherlands.

**Keywords:** acute HIV infection, cohort, early HIV infection, HIV, phylogeny, transmission

## Abstract

Timely identification of acute or early HIV infection (AEHI) is important to help prevent onward transmission, and understanding the number of secondary infections resulting from individuals with AEHI is key to planning HIV prevention services and case finding.

**Design:**

We performed a phylogenetic investigation of a dense sample of individuals with AEHI who took part in the Netherlands Cohort Study on Acute HIV infection (NOVA) in the Netherlands during 2015-2021.

**Methods:**

Transmission clusters were identified using phylogenetic analyses based on HIV pol sequences. The Tamura-Nei model was used to estimate genetic distance. A number of 1000 bootstraps was used to check the reliability of clustering using maximum likelihood. A cluster was defined as having a bootstrap value of at least 95% and a genetic distance of at most 1.5%. Sensitivity analyses using different values for the bootstrap and genetic distance were performed to study the reproducibility of the clustering.

**Results:**

Of the 156 participants included in NOVA between July 2015 and April 2021, 134 individuals for whom baseline characteristics and genotypic resistance data at baseline were available could be included. We identified 10 clusters, but the majority of persons (111/134) were not part of a cluster, suggesting mainly independent transmission events.

**Conclusion:**

Mainly independent transmission events among a study population consisting predominantly of MSM in a low-incidence high-resource setting is likely the result of active AEHI case finding and direct start of treatment, and the roll-out over recent years of preventive measures such as preexposure prophylaxis.

## Introduction

The acute stage of HIV infection is a brief, but highly infectious phase of infection [1]. Individuals who are unaware of their status may transmit HIV to multiple individuals during this infectious phase. Timely identification of acute or early HIV infection (AEHI) (here defined as the first 6 months after infection) is therefore important to help prevent onward transmission. Understanding the number of secondary infections due to AEHI is also important, for the planning of HIV prevention services, such as immediate treatment initiation and preexposure prophylaxis (PrEP), and for case finding. Previous studies in low-incidence settings, conducted before an approach of ‘test and treat’ was widely advocated, described substantial clustered transmission during AEHI [2–6]. Mathematical modelling studies on the proportion of new HIV infections attributable to AEHI cases varied considerably, with estimates ranging from below 10% to over 80% [7–9]. To understand the more contemporary transmission dynamics during AEHI, we performed a phylogenetic analysis among 134 therapy-naive persons with AEHI participating in the Netherlands Cohort Study on Acute HIV infection (NOVA) in the Netherlands between July 2015 and April 2021 [10].

## Methods

The NOVA is a cohort study that prospectively includes adults diagnosed with AEHI who are willing to start combination antiretroviral therapy (cART) within 24 h of enrolment [10]. AEHI is defined as the first 6 months after infection according to Fiebig staging based on the detection of plasma HIV-RNA by RT-PCR, HIV p24 antigen and anti-HIV antibodies by fourth generation ELISA, and western blot [11]. Participants diagnosed in Fiebig VI could only be included if a negative HIV test less than 6 months prior to their diagnosis was performed. A sequence analysis to find genotypic resistance of HIV was performed using viral sequences from the participants using the Stanford HIV Drug Resistance database version 9.0 [12]. The HIV subtype was identified using the REGA subtyping tool [13]. Clinical and demographic data were collected in collaboration with Stichting HIV Monitoring (SHM, HIV Monitoring Foundation), which coordinates the AIDS Therapy Evaluation in the Netherlands (ATHENA) cohort, a nationwide HIV cohort, which prospectively captures data of approximately 98% of all people with HIV (PWH) in care [14]. Clinical data in the ATHENA cohort are collected by trained data monitors using standardized data collection tools. Currently, the nine HIV treatment centers that participate in the NOVA serve 65% of all PWH in care in the Netherlands, and cover the areas known to have the highest HIV prevalence [15].

Transmission clusters were identified using phylogenetic analyses based on HIV pol sequences. This involved either protease (PRO)–reverse transcriptase (RT)–integrase (INT) (*n* = 26), PRO-RT (*n* = 56), or reverse transcriptase (*n* = 52). The HIV-1 pol sequences were aligned to 53 reference sequences from known subtypes from group M (subtypes A–K and circulating recombinant forms). For each of the participants’ sequences, 10 mostly similar sequences were retrieved from GenBank and added to our dataset. All sequences were aligned and trimmed to equal length. After removal of duplicate sequences from GenBank and one erroneous NOVA sequence this resulted in 941 sequences (53 Los Alamos, 133 NOVA, 755 GenBank) with a length of 1319 nucleotides. As INT was not always sequenced, the phylogenetic analysis included only PRO-RT. The analysis was based on the maximum likelihood method using the Tamura-Nei model to estimate genetic distance. A number of 1000 bootstraps was used to check the reliability of the clustering. A cluster was defined as having a bootstrap value of at least 95% and a genetic distance of at most 1.5%. Sensitivity analyses using different values for the bootstrap and genetic distance were performed to study the reproducibility of the clustering.

The NOVA was approved by the medical ethical committee of the Amsterdam University Medical Centers (Academic Medical Center site) (NL51613.018.14) and all participants provided written informed consent.

## Results

Of the 156 participants included in the NOVA between July 2015 and April 2021, 134 individuals for whom baseline characteristics and genotypic resistance data at baseline were available could be included in the phylogenetic analysis. Table 1 presents an overview of the baseline characteristics. Most of the participants acquired HIV through sex with other men (*n* = 117; 87.3%), similar to the majority of new HIV diagnoses in the Netherlands [10]. Diagnoses were predominantly in Fiebig stage IV–VI (*n* = 95; 70.9%), and most individuals were infected with HIV-1 subtype B (*n* = 98; 73.1%). Individuals had high plasma viral loads at enrollment with a median HIV-RNA of 5.8 (IQR 4.7–6.7) log_10_ copies/ml. The median CD4^+^ T-cell count at baseline was 510 (IQR 355–683) cells/μl, and CD4^+^ T-cell counts of below 200 cells/μl were uncommon (*n* = 4; 3.0%, data not shown). Participants who were enrolled in Amsterdam accounted for two-third of all NOVA participants (*n* = 86; 64.2%). Over one-fourth of all participants (*n* = 37; 27.6%) were enrolled in the region of Rotterdam.

**Table 1:** Baseline characteristics of 134 NOVA cohort study participants.

	*n* or median	Percent or IQR
Age in years	36	28–47
Gender		
Male	130	97.0%
Female	4	3.0%
Region of birth^a^		
Europe	103	77.4%
Caribbean	10	7.5%
South America	10	7.5%
Other	10	7.5%
MSM		
Yes	117	87.3%
No	6	4.5%
Unknown	11	8.2%
Site of HIV diagnosis		
STI clinic	65	48.5%
General practice	39	29.1%
Hospital	18	13.4%
Other^b^	8	6.0%
Unknown	4	3.0%
Subtype		
B	98	73.1%
Non-B	36	26.9%
*A*	10	
*CRF02_AG*	7	
*C*	6	
*CRF01_AE*	5	
*F*	4	
*CRF06_cpx*	2	
*CRF47_BF*	1	
*G + CRF02_AG*^c^	1	
Fiebig stage		
I	3	2.2%
II	17	12.7%
III	5	3.7%
IV	37	27.6%
V	39	29.1%
VI^d^	19	14.2%
Unknown	14	10.4%
Plasma viral load (log10 copies/ml)^e^	5.8	4.7–6.7
CD4^+^ T-cell count (cells/μl)^f^	510	355–683
CD8^+^ T-cell count (cells/μl)^g^	941	550–1485
CD4^+^/CD8^+^ T-cell ratio^h^	0.5	0.4–0.9

ART, antiretroviral therapy; IQR, interquartile range; STI; sexually transmitted infection.

a 1 value missing.

b Community-based testing (*n* = 4), own initiative (*n* = 4).

c G + CRF02_AG was based on a best match (4.52%) with this subtype according to the Stanford University HIV Drug Resistance Database [12].

d Participants diagnosed in Fiebig VI could only be included in case of a negative test result available in the previous six months.

e Four values missing.

f Four values missing.

g Five values missing.

h Five values missing.

The analysis showed 10 clusters, eight of which included two NOVA participants, and two of which included three or more persons (Table 2). In total, 111 participants with AEHI were not part of a cluster. Apart from one cluster involving HIV-1 subtype CRF02_AG, all clusters were of subtype B. Of the 23 participants found to be part of a cluster, 12 (52.2%) enrolled in the region of Amsterdam. Over one-third of the 23 participants involved in clusters (*n* = 8; 34.8%) were enrolled in the region of Rotterdam. In 8 of 10 clusters found, participants enrolled in study sites in the same city. One cluster involved three participants from the same city who were diagnosed within 4 months from each other. The largest cluster involved four participants diagnosed in Fiebig 1, 5 and 6, all of whom started cART within 8 days from the date of their diagnosis, and three of whom were diagnosed in the same city over a period of 3 years. In 8 of 10 clusters, individuals received their diagnosis within a maximum of 6 months apart. Sensitivity analyses using different values for the bootstrap and genetic distance did not affect the clustering found in this study.

**Table 2:** HIV transmission clusters between 2015 and 2021 involving Netherlands Cohort Study on Acute HIV infection participants and their baseline characteristics.

Sex	Subtype	HIV diagnosis	Region enrolled	Fiebig at diagnosis	CD4^+^ count at diagnosis	Plasma HIV-RNA at diagnosis	Mutations at baseline	Drug class
Male	B	09/2017	Rotterdam	F6	700	22 600	T215S	NRTI
Male	B	07/2019	Rotterdam	F5	300	19 400 000	T215S	NRTI
Male	B	08/2019	Rotterdam	F2	270	33 100 000	T215S	NRTI
Male	B	11/2019	Amsterdam	Unknown	808	2 263 455	T215S	NRTI
Male	B	10/2015	Amsterdam	F4	530	84 675	None	None
Male	B	12/2015	Amsterdam	F6	590	33 862	None	None
Male	B	10/2018	Amsterdam	F2	580	8 356 721	None	None
Male	B	04/2019	Amsterdam	F3	340	10 000 000	None	None
Male	B	10/2018	Rotterdam	F4	200	10 000 000	None	None
Male	B	12/2018	Rotterdam	F5	1200	46 700	None	None
Male	B	06/2018	Amsterdam	F5	750	4379	None	None
Male	B	03/2018	Amsterdam	F5	530	69 028	None	None
Male	B	03/2016	Amsterdam	F5	250	1 420 000	None	None
Male	B	08/2016	Amsterdam	F1	510	98 700	None	None
Male	B	03/2019	Amsterdam	F6	270	1 195 913	None	None
Male	B	08/2017	Leiden	F5	269	41 400	None	None
Male	B	08/2017	Leiden	F5	438	16 000	None	None
Male	B	01/2018	Leiden	F6	680	88 400	None	None
Male	B	10/2016	Amsterdam	F5	470	650 000	None	None
Male	B	04/2017	Amsterdam	F6	705	775 502	None	None
Male	CRF02_AG	05/2017	Rotterdam	F5	270	1 060 000	None	None
Male	CRF02_AG	09/2017	Rotterdam	F5	420	376 000	None	None
Male	CRF02_AG	08/2017	Rotterdam	F6	570	43 300	None	None

Participants diagnosed in Fiebig VI could only be included in case of a negative test result available in the previous 6 months; CD4^+^ T-cell counts are presented in cells/μl; HIV-RNA is presented in RNA copies/ml. NRTI, nucleoside reverse transcriptase inhibitors.

Transmitted drug resistance mutations found were not relevant to PrEP available in the Netherlands or current cART options, in line with the low and stable prevalence of transmitted drug resistance among the general population with HIV in the Netherlands [15]. Among the 26 participants sequenced for integrase, no mutations associated with resistance against integrase strand transfer inhibitor were found (data not shown).

## Discussion

Testing, immediate treatment and preventive measures for HIV are interventions that require major public health commitment. A better understanding of spatiotemporal clustering during AEHI can provide information about transmission networks in specific populations and as such can help make evidence-informed decisions about targeted prevention measures for distinct demographic groups in specific regions. In this large sample of persons with AEHI in the Netherlands, we found multiple small AEHI clusters in mostly separate geographic locations, often occurring within 6 months, but the majority of participants (111/134) were not part of a cluster. This suggests that transmission during AEHI among MSM during our study period largely resulted from independent onward transmission events, rather than from large clustered outbreaks. This implies that these independent transmission events likely represent transmission from an index case with a still undiagnosed HIV infection, or from someone on ART who is not virally suppressed. Concerning the former, identifying such individuals may benefit from optimized partner notification, availability of self-testing as well as enhanced surveillance by medical specialists in hospitals, at local sexual health clinics and by general practitioners. Emphasis should be placed on the extension of strategies to test and immediately treat acute HIV infection that have proven to be effective in a Dutch setting [16].

By comparison, most [2–5] but not all [17] previously reported studies in similar settings did describe substantial clustered transmission during AEHI. Importantly, these studies were conducted at a time when immediate start of cART following diagnosis, improved diagnosis of AEHI, and the roll-out of PrEP, were not yet widely implemented. These factors likely contributed to the reduced clustering of infections during AEHI, which we observed in the NOVA, which is a more recently established prospective cohort study. In terms of public health commitment, the findings of this study, therefore, underline the importance of continuing such preventive efforts. However, if transmission events during AEHI are indeed mainly independent, this would preclude the opportunity of concentrating public health efforts on specific networks, and would support spending of public health resources on overall high PrEP coverage of at-risk populations.

A strength of this analysis is that this study sample is likely to be representative of the Dutch HIV epidemic, as we included approximately one-fourth of all individuals diagnosed with AEHI in the Netherlands during the overlapping study inclusion period [18]. However, as we did not systematically sequence the general larger HIV population, we were not able to assess the contribution of AEHI to overall HIV transmission occurring in the Netherlands between 2015 and 2021. Moreover, the small clusters that we found in this study may have contained individuals diagnosed with AEHI who did not take part in the NOVA and whose sequence data could, therefore, not be included, or persons diagnosed outside AEHI who could thus not take part in the NOVA, or undiagnosed individuals.

In conclusion, in this study population consisting predominantly of MSM in a low-incidence high-resource setting, transmission among individuals with AEHI appeared to largely result from independent transmission events rather than large outbreaks resulting from a single AEHI index case. This limited clustering of AEHI cases that we observed is likely the result of active AEHI case finding and direct start of cART, as well as the roll-out over recent years of preventive measures such as PrEP, and provides hope that with such efforts we can eliminate linked transmissions and achieve a future free of HIV transmission.

## Acknowledgements

We would kindly like to thank all participants of the NOVA cohort study. We thank Ellsworth Campbell and Bill Switzer from the CDC and Dmitry Kireev for their kind help with the analysis. We would also like to thank the members of the Erasmus MC HIV Eradication Group (EHEG), all medical doctors, research nurses, and laboratory personnel involved in the NOVA cohort study at the Amsterdam University Medical Centre, the Erasmus University Medical Centre, Maasstad Hospital Rotterdam, Onze Lieve Vrouwe Gasthuis, DC Klinieken, UMC Utrecht, Radboud UMC, Leiden UMC, and Rijnstate Hospital for enrolment, follow up and (handling of) sampling of NOVA participants, including Karin Grintjes, Reinout van Crevel, Janette Rahamat-Langendoen, Henk Scheper, Jutte de Vries, Annouschka Weijsenfeld, Frank Pijnappel, Agnes Harskamp, Neeltje Kootstra, Suzanne Jurriaans, Marc van der Valk, Hans-Erik Nobel, Arne van Eeden, Loek Elsenburg, Kees Brinkman, Imke Hooijenga, Janneke Stalenhoef, Mark Claassen, Petra van Bentum, Jan den Hollander, Jannigje Smit, Yvonne Muller, Peter Katsikis, Tokameh Mahmoudi, Rob Gruters, Andy Hoepelman, Monique Nijhuis, Anne Wensing. We would also like to thank Stichting HIV Monitoring for providing data collection and management support, including Mariska Hillebregt, and Leonie de Groot – Berndsen.

Author contributions: H.P., C.R., A.V., J.v.K. and D.v.d.V. designed the phylogenetic study. A.v.S. and J.v.K. provided baseline and sequence data. H.P. and D.v.d.V. performed the study, analyzed the data, and wrote the first draft. H.P., C.R., A.V., A.v.S., G.d.B., M.D., J.P., P.R., J.v.K., and D.v.d.V. interpreted the results and critically revised subsequent versions of the manuscript. All authors approved the final version of the manuscript.

Funding: the NOVA is part of the H-TEAM Initiative, which is supported by Aids Fonds (grant number: 2013169); Amsterdam Dinner Foundation, Bristol-Myers Squibb International Corp (study number: AI424-541); Gilead Sciences Europe Ltd (grant number: PA-HIV-PREP-16-0024); Gilead Sciences (protocol numbers: CO-NL-276-4222, CO-US-276-1712); Janssen Pharmaceuticals (reference number: PHNL/JAN/0714/0005b/1912fde); M.A.C. AIDS Fund, ViiV Healthcare (PO numbers: 3000268822, 3000747780); and ZonMw (grant number: 522002003). The ATHENA cohort is managed by Stichting HIV Monitoring and supported by a grant from the Dutch Ministry of Health, Welfare and Sport through the Centre for Infectious Disease Control of the National Institute for Public Health and the Environment.

### Conflicts of interest

The following authors have received grants and honoraria: G.d.B. from Bristol-Meyer Squibbs, Gilead Sciences, ViiV Healthcare, Exevir, Aidsfonds and Takeda, all paid through institution; P.R. from Gilead Sciences, Janssen Pharmaceutica, ViiV Healthcare and Merck, Gilead Sciences, all paid to his institution; C.R. from AIDSfonds, ZonMW, Dutch Federation Medical Specialists, Health Holland, Merck, Janssen-Cilag, Gilead and ViiV Healthcare; J.v.K from NIH, ZonMW, Health Holland, and Aidsfonds. All other authors declared no conflicts of interest.
